# Are women with disabilities less likely to utilize essential maternal and reproductive health services?—A secondary analysis of Pakistan Demographic Health Survey

**DOI:** 10.1371/journal.pone.0273869

**Published:** 2022-08-29

**Authors:** Shafaq Mahmood, Waqas Hameed, Sameen Siddiqi

**Affiliations:** Department of Community Health Sciences, Aga Khan University, Karachi, Pakistan; Jhpiego, UNITED STATES

## Abstract

**Background:**

People with disabilities deal with widespread exclusion from healthcare services, including sexual and reproductive health (SRH) rights. Studies analyzing the relationship between disability and key SRH utilization outcomes have often reported mixed findings. In Pakistan, very little to no literature is available on this topic, therefore we aim to determine inequalities in the utilization of essential maternal and reproductive health services between women with and without disabilities in Pakistan.

**Methods:**

This was a secondary analysis of Pakistan Demographic Health Survey 2017–18 performed on a weighted sample of 6,711 women aged 15–49 years with a live birth in the 5 years preceding the survey. Six types of disabilities were assessed i.e. vision, hearing, communication, cognition, walking and self-care. Utilization of essential maternal and reproductive health services was assessed through a set of four outcome variables: (i) modern contraceptive use; (ii) skilled antenatal care (ANC); (iii) skilled birth attendance (SBA); and (iv) skilled postnatal care (PNC). Multivariate Cox regression analysis was performed to determine the association between dependent and independent variables. Data were analyzed using Stata MP Version 16.0.

**Results:**

A total of 6,711 women were included out of which 14.1% (n = 947) live with at least one form of disability. Mean age was 29.4 (S.E = 0.13) years. The most prevalent form of disability was vision (7.0%), followed by walking (4.8%), cognition (4.8%) and hearing (1.8%). Women with disabilities were comparatively less educated, belonged to older age group, and had higher parity than their non-disabled counterparts. With the exception of modern contraceptive use, which was more prevalent in the group with disabilities, women with disabilities were less likely to utilize skilled ANC, SBA and PNC in bivariate analysis. However, these associations turned insignificant in the adjusted model. Overall, no statistically significant differences were observed in the utilization of essential reproductive health services between women with and without disabilities after adjusting for important covariates.

**Conclusion:**

Our analyses did not find any statistically significant differences in the utilization of essential maternal and reproductive health services between women with and without disabilities. In-depth research utilizing qualitative or mixed methods is required to understand how well the healthcare system in Pakistan is responsive to the different needs of disabled women.

## Introduction

Providing populations with disabilities an equitable chance to exercise a full range of their social, political, economic, and civil rights has become a major global concern in recent years. The world has witnessed a dramatic rise in the prevalence of disabilities due to an inevitable surge in chronic health conditions [1]. Globally, more than one billion people on earth are living with some form of disability, with nearly 80% of them disproportionately located in Low- and Middle- Income Countries (LMICs) [1]. By definition, disability encompasses any long-term physical, mental, cognitive, or sensory impairment that in combination with other factors restricts a person’s ability to participate effectively in society as compared to others [2].

Global commitments such as Sustainable Development Goals (SDGs) and the United Nations Convention on the Rights of Persons with Disabilities (UNCRPD) have laid profound emphasis on increasing the breadth of affordable and quality ‘health for all’, with a special focus on socially marginalized groups [3, 4]. However, research has indicated that apart from fewer educational and vocational opportunities, people with disabilities also deal with widespread exclusion from healthcare services, including sexual and reproductive health (SRH) rights [5–7]. Barriers such as physically inaccessible healthcare centers, lack of specialized transport facilities, stigma and discrimination at the point of healthcare delivery, and inadequate training and skills of healthcare workers (HCWs) to cater for their different needs may limit the utilization of health services by people with disabilities despite their greater need [8].

According to the United Nations Population Fund (UNFPA) report on the promotion and protection of the rights of persons with disabilities, women with disabilities in several parts of the developing world are denied their basic SRH rights [9]. Societal norms and the perceptions of inadequacy (both physical and mental) often precludes people with disabilities from having a fair access and usage of SRH services [10]. A qualitative survey from Northern Uganda exploring the barriers and intersecting discrimination faced by the people with disabilities while accessing SRH services found that the utilization of SRH services among people with disabilities is shaped by multiple concurrent intersections of gender, disability, and violence. Women in particular were reported to be discriminated against the mainstream society (by being denied the full expression of their SRH rights such as getting married or using contraception), and were exposed to violence, stigmatization and discrimination by the healthcare providers and the community members [11]. Likewise, another study examining the relationship between disability and the use of SRH services in association with other social determinants of health also highlighted the role of societal power structures in preventing women with disabilities from using the SRH services at the same frequency as non-disabled woman [12]. Falling victim to the widely held misconception of being less capable of sexual activity and childbearing, women with disabilities are largely ignored in reproductive health information and services [13]. Some of the key challenges faced by women with disabilities in the literature include; difficulty accessing the information on common SRH issues, limited or no family planning choices, forced sterilization and abortion, poorly managed pregnancy and birth, intimate partner violence and little to no right for informed decision making [14–16]. Consequently, women with disabilities are at a higher risk of developing pregnancy and birth related complications such as low birth weight, preterm deliveries, fetal growth restriction, and increased incidence of operative delivery [17]. Moreover, estimates of maternal mortality and morbidity in LMICs are also higher among socially marginalized groups and women with disabilities [18, 19].

Pakistan, the fifth most populous country in the world, also has one of the highest maternal mortality rate (MMR) in the region (186 per 100,000 live births) [20, 21]. According to the recent Pakistan Demographic and Health Survey (PDHS 2017–18), 15% of the women of reproductive age group currently lives with at least one type of disability [22]. Although, the country has undergone several health system reforms to increase the coverage and accessibility of healthcare services, it is still included in the top ten countries with the most inequitable healthcare interventions [23]. A study assessing the healthcare access for people with physical disabilities in rural Punjab reported poor arrangements and discriminatory attitudes towards both male and female patients with disabilities. In addition, women with disabilities also expressed extreme dissatisfaction with the quality of services and information being provided by healthcare professionals regarding SRH issues [24].

Considering the global implication of this issue, a growing number of researchers have attempted to study the utilization patterns of SRH services and rights among groups with disabilities. However, studies analyzing the relationship between disability and key SRH utilization outcomes have often reported mixed findings [25–27]. In order to develop a better understanding of this subject, the following study aims to determine inequalities in the utilization of essential maternal and reproductive health services between women with and without disabilities in Pakistan.

## Methodology

### Data

This paper is based on a secondary analysis of most recent and nationally representative Pakistan Demographic and Health Survey (PDHS 2017–18) dataset. The PDHS provides updated and reliable information on several demographic and health indicators such as contraception, maternal and child health issues, nutrition, housing characteristics, women empowerment, domestic violence, HIV awareness and knowledge, and disability. The inferences drawn through PDHS data are utilized for planning and implementation of public health programs at the federal and provincial government levels.

### Sample design

The PDHS 2017–18 utilized a two-stage stratified sample design using most recent census frame. A total of 8 regions were stratified into urban and rural residence–yielding 16 strata. Sample from each strata were selected through a two-staged selection procedure. In the first stage, 580 clusters (enumeration blocks) were randomly selected from each stratum using probability proportional to size technique. In the second stage, 28 households from each cluster were systematically sampled through a household listing.

### Study population

A total of 15,068 ever-married women aged 15–49 years were interviewed. As a standard practice, PDHS collects data for antenatal care (ANC), and skilled birth attendance (SBA) for preceding five years, whereas for postnatal care (PNC), the sample is restricted to women with a live birth in last 2 years. Based on this criteria, the final analysis was performed on an unweighted sample of 8,287 women aged 15–49 years (weighted sample 6,711) who had a live birth in the 5 years before the survey. Whereas, for PNC the analysis was performed on a weighted count of 3,935 women.

### Measures

#### Outcome variables

Utilization of essential maternal and reproductive health services was assessed through a set of four key outcome variables: (i) modern contraceptive use; (ii) skilled antenatal care (ANC); (iii) skilled birth attendance (SBA); and (iv) skilled postnatal care (PNC).

The modern contraceptive methods included: female and male sterilization; oral contraceptive pills (OCPs); intrauterine contraceptive device (IUCD), male condoms; injectables; lactational amenorrhea method (LAM); and standard days method (SDM). The variable for current use of modern contraceptives was dichotomized and coded as ‘1’ if women used any of the above listed modern methods, and ‘0’ otherwise.

Similarly, the other three outcome variables i.e. skilled ANC; SBA; and skilled PNC were also coded as binary, where ‘0 = care received from unskilled provider’ and ‘1 = care received from skilled provider”. The classification of skilled healthcare professionals is country specific and the PDHS typically includes doctor, nurse, midwife, lady health visitor (LHV) or community midwife as skilled providers, and the same was considered true for this study.

Since the data in PDHS is more consistently available for most recent birth of women, hence we performed the analysis for all four outcome variables for the last live birth of women respondent before the survey year.

#### Independent variable

The DHS program’s disability module has been developed in collaboration with the Washington Group on Disability Statistics (WG) and the U.S. Agency for International Development (USAID). The tool was originally developed by the WG using the WHO’s International Classification of Functioning, Disability and Health (ICF) as a conceptualizing framework [28].

The module covers six comprehensive disability domains: vision, hearing, communication, cognition, walking and self-care. Each functional domain has four responses: 0 = no difficulty, 1 = some difficulty, 2 = a lot of difficulty, and 3 = ‘cannot function at all’ in the specified domain.

We created a dichotomous variable coded as ‘1’ if the women reported at least “some difficulty” in one or more domains and ‘0’ for ‘no difficulty’ in all six domains. Additionally, a separate binary variable for each functional domain was also generated and coded as ‘0’ if women reported no disability and ‘1’ if she reported at least some difficulty in that specified domain.

An additional variable was generated to measure “severe disability” status of the study population. For this, any women who reported experiencing “a lot of difficulty” or “cannot function at all” in any of the six domains was categorized as having “severe disability status” and coded as ‘1’ and ‘0’ otherwise [28].

#### Covariates

The analysis also accounted for different covariates that could serve as potential confounders in the association between disability status and essential maternal and reproductive health outcomes. This included age of the respondent (15–24, 25–34 and 35–49 years), education level (no education, primary, secondary, and higher), wealth index (poor and rich) type of residence (urban/rural), and parity (0–1, 2–3, and >3).

#### Effect modifiers

We also performed a sub-group analysis to see if the relationship between women’s disability status and key maternal and reproductive health outcomes varied by her wealth status (poor vs. rich) and type of residence (rural vs. urban). The wealth index originally computed by the DHS program using principal component analysis has five quintiles (poorest, poorer, middle, richer and richest). For this analysis, we needed a binary measure for wealth index that is 60% poorest and 40% richest. Therefore, the variable was recoded by merging “poorest, poorer and middle” into one category coded as ‘0 = poor’ and “richer and richest” merged into ‘1 = rich’. The reconstruction of wealth index was done after consulting with DHS experts. Previous studies using DHS data sets have also adapted this methodology [29, 30].

### Statistical analysis

Data were analyzed in three stages. In the first stage, descriptive analysis was performed to describe the socio-demographic profile of women with and without disabilities using frequencies, percentages, means and standard deviation. Furthermore, Pearson’s chi-square was applied to compare the socio-demographic profile of the two groups. We also estimated the prevalence of disability overall and separately for each functional domain.

In the third stage, crude and adjusted prevalence ratio (APR) along with 95% confidence intervals (CIs) were calculated using multivariate Cox regression analysis, keeping the time variable constant [31]. The relationship between disability status and reproductive health outcomes was explored separately across each functional domain of disability. The multivariate model was also adjusted for other confounder variables such as age, education, wealth index, parity and type of place of residence. Lastly, sub-group analysis was conducted to assess the role of women’s wealth status and type of residence as effect modifiers in the relationship between overall/severe disability status and reproductive health outcomes.

Data were analyzed using Stata MP Version 16.0 (StataCorp LP, College Station, TX, US). All analyses accounted for complex survey design, strata, primary sampling units (clusters), and probability sampling using women’s individual sample weights. P-values of <0.05 were considered significant.

### Ethics statement

This study is based on a secondary data that is publicly available on DHS website (https://dhsprogram.com/data/available-datasets.cfm), therefore, an informed consent and ethical approval was not required for this study. However, the data access was formally gained on request from MEASURE DHS.

## Results

### Socio-demographic profile of study sample

Table 1 compares the socio-demographic characteristics of women with and without disabilities who had a recent live birth in the 5 years preceding the survey. Of 6,711 women, 14.1% (n = 947) had at least one or more disabilities.

**Table 1:** Sample analytical profile, Pakistan (PDHS 2017–18).

Background characteristics	Disabled		Non-disabled		Overall	
	n	%	n	%	N	%
**Age (years)**						
15–24	117	12.3	1429	24.8	1545	23.0
25–34	484	51.1	3241	56.2	3725	55.5
35–49	346	35.6	1094	19.0	1440	21.5***
Mean ± SE	29.4 ± 0.13					
**Education**						
No education	437	46.2	2775	48.1	3212	47.9
Primary	186	19.6	911	15.8	1097	16.3
Secondary	234	24.7	1258	21.8	1492	22.2
Higher	90	9.5	821	14.2	911	13.6*
**Wealth index** ^**1**^						
Poor	613	64.7	3501	60.8	4115	61.3
Rich	334	35.3	2263	39.2	2597	38.7
**Residence**						
Urban	336	35.5	1912	33.2	2248	33.5
Rural	611	64.6	3852	66.8	4463	66.4
**Parity**						
0–1	115	12.1	1222	21.2	1337	19.9
2–3	274	29.0	2297	39.8	2571	38.3
>3	558	58.9	2245	39.0	2803	41.8***
**Total**	**947**	**100**	**5764**	**100**	**6711**	**100**

*** <0.001 ** <0.01 * <0.05

^1^Poor includes “poorest”, “poorer” and “middle” category, while rich includes “richer” and richest”

Mean age of women was 29.4 (S.E = 0.13) years, with majority of them (55.5%) falling between 25–34 years. Nearly half (48.0%) of them had received no formal education. Approximately two-third of the study sample belonged to the poor socioeconomic class and lived in rural areas i.e. 61.3% and 66.4% respectively. Overall, one fifth of the study sample had less than two living children.

As compared to the non-disabled women (19.0%), a significantly higher proportion (35.6%) of women with disabilities belonged to the older age group (35–49 years). In addition, the non-disabled women were more likely to acquire education up to higher level as compared to the women with disabilities (14.2% vs. 9.5%). A significantly greater proportion of women with any form of disability had more than 3 living children (58.9%) as compared to the non-disabled group (39.0%). No significant difference in the socio-economic status and type of residence was seen among the two groups.

### Prevalence of disability by functional domains

**Fig 1:**
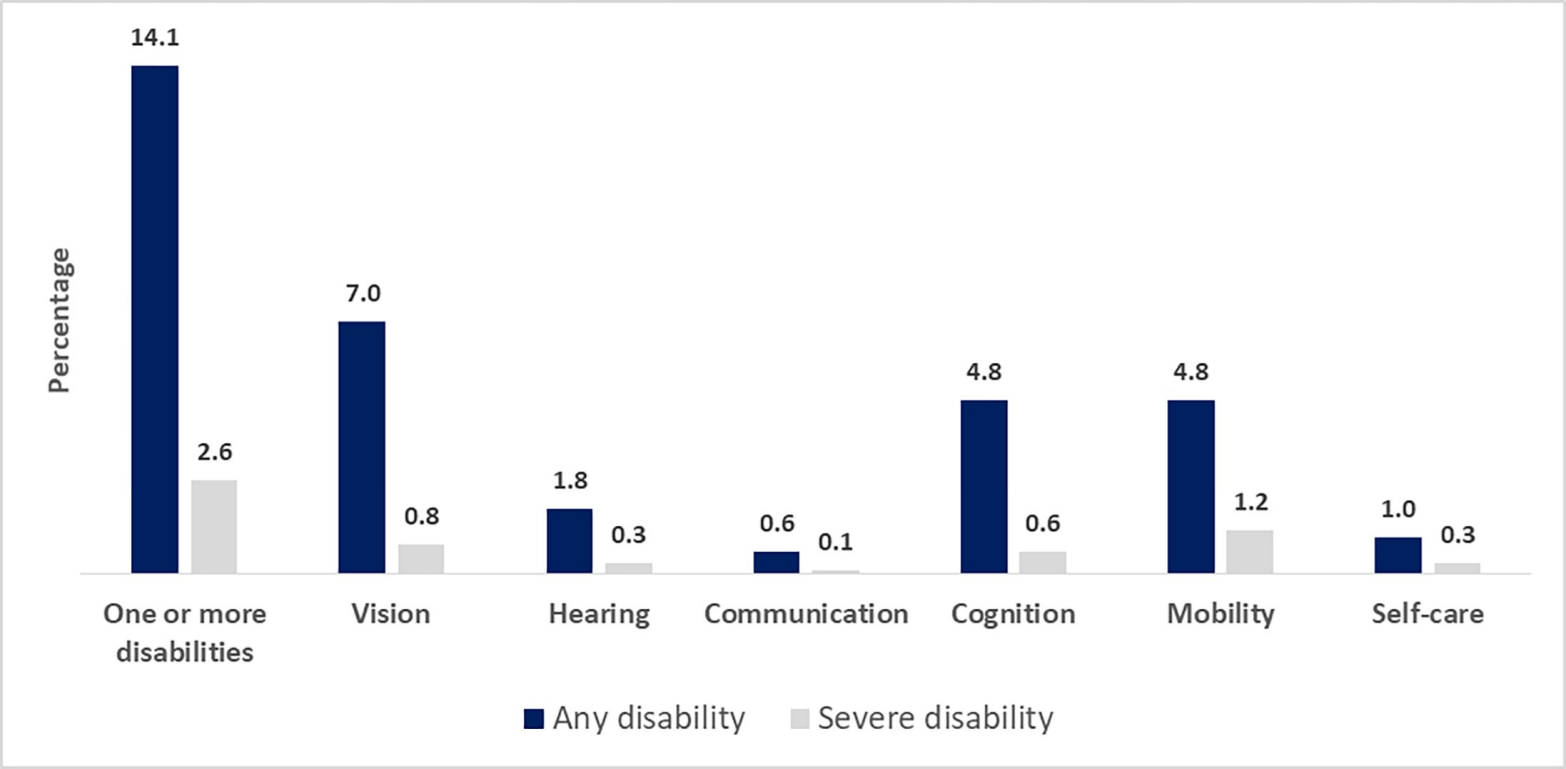
Prevalence of any disability and severe functional disability among women aged 15–49 years with a live birth in the 5 years before the survey, according to the domains (n = 6,711).

Fig 1 shows the distribution of disabilities across each functional domain by ‘any’ and ‘severe’ disability status. Overall, 14.1% women had disability in one or more domains, whereas 2.6% had any form of “severe” disability. The most prevalent form of disability was vision (7.0%), followed by walking (4.8%), cognition (4.8%) and hearing (1.8%), whereas difficulty in self-care and communication was only reported by 1.0% and 0.6% of the participants, respectively. Regarding severe disability, the most commonly reported domain was of walking (1.2%).

### Utilization of essential maternal and reproductive health services by disability status

**Fig 2:**
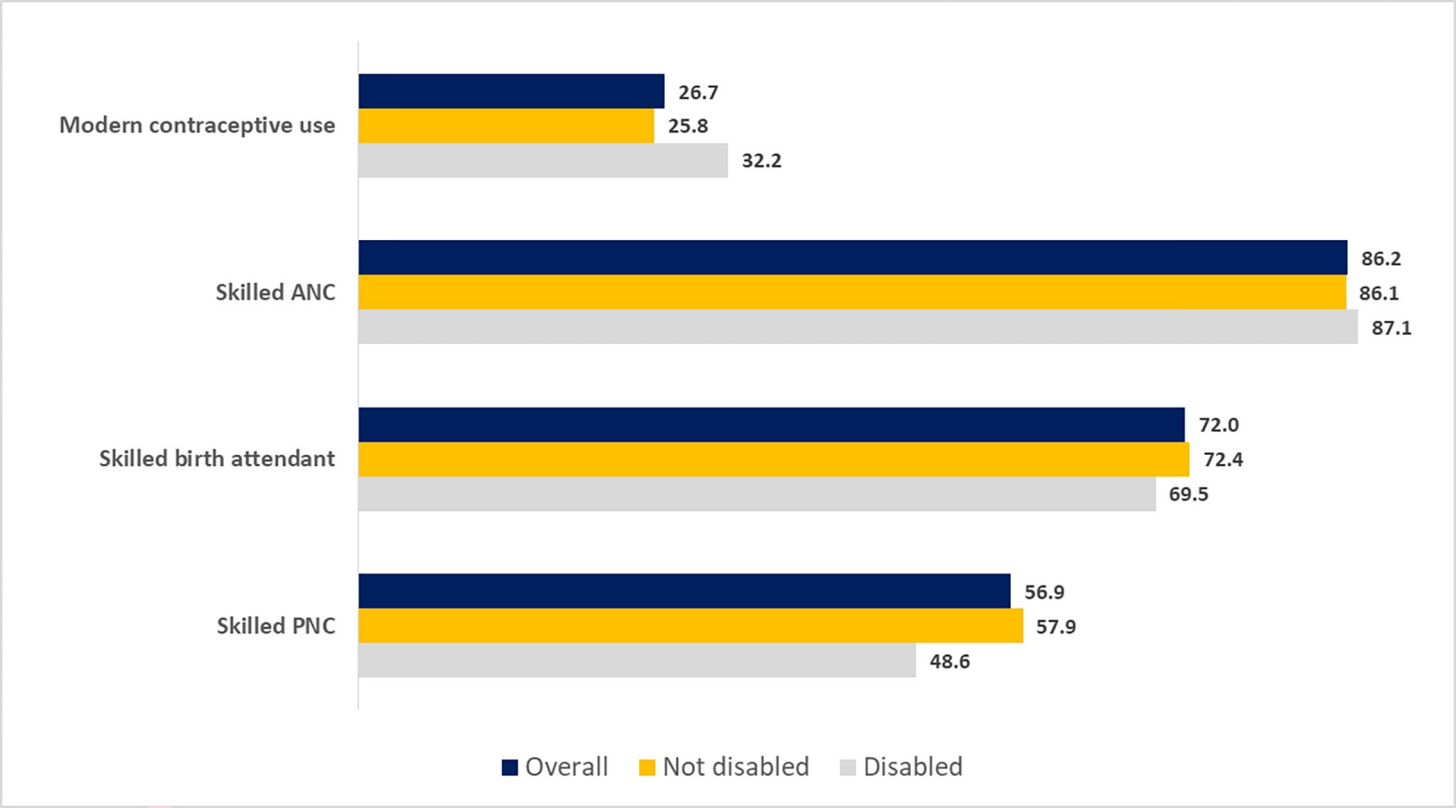
Utilization of essential maternal and reproductive health services by women aged 15–49 years with a live birth in the 5 years before the survey, according to the disability status. (n = 6,711). Note: For skilled PNC, the study sample includes women aged 15–49 years giving birth in the 2 years preceding the survey. (n = 3,935).

Fig 2 demonstrates the distribution of essential maternal and reproductive health services by disability status of the women. Overall the prevalence of modern contraceptive use, skilled ANC, SBA, and skilled PNC among the study population was 26.7%, 86.2%, 72.0% and 56.9%, respectively.

It was seen that the modern contraceptive usage was comparatively higher among the group with disabilities (32.2% vs. 25.8%). On the other, SBA and skilled PNC were less prevalent in women who had some disability in one or more functional domains. The results of skilled ANC were almost comparable among the two groups.

#### Findings of regression analysis

Table 2 shows the crude (PR) and adjusted prevalence ratio (APR) along with 95% Confidence Intervals (CIs) for assessing inequalities in the utilization of essential reproductive health services between women with and without disabilities. In the crude model, women with any form of disability were 1.25 times more likely to use modern contraceptives as compared to the non-disabled women (PR = 1.25; CI 1.08–1.45; p value <0.01). Similarly, women who had disabled vision, cognition and walking had higher odds of using modern contraceptives in the crude model (p value <0.05). However these associations turned insignificant in the adjusted model.

**Table 2:** Crude (PR) and adjusted^1^ prevalence ratio (APR) along with 95% CI for inequalities in the utilization of essential reproductive health services between women with and without disabilities. (n = 6,711).

Independent variables	Outcome variables											
	Modern contraceptive use			Skilled ANC			Skilled birth attendant			Skilled PNC		
	n (%)	PR (95% CI)	APR (95% CI)	n (%)	PR (95% CI)	APR (95% CI)	n (%)	PR (95% CI)	APR (95% CI)	n (%)	PR (95% CI)	APR (95% CI)
**No severe disability (Ref)**	1754 (26.8)	1	1	5636 (86.2)	1	1	4717 (72.2)	1	1	2208 (57.2)	1	1
**Any severe disability in one or more domains**	38 (21.7)	0.81 (0.54–1.22)	0.75 (0.50–1.13)	149 (86.1)	1.0 (0.91–1.1)	1.03 (0.95–1.12)	115 (66.5)	0.92 (0.80–1.06)	0.98 (0.87–1.12)	29 (40.0) *	0.70 (0.46–1.05)	0.83 (0.55–1.22)
**No disability (Ref)**	1486 (25.8)	1	1	4960 (86.1)	1	1	4175 (72.4)	1	1	2026 (58.0)	1	1
**Any disability in one or more domains**	305 (32.2) **	1.25 (1.08–1.45) **	1.10 (0.94–1.28)	825 (87.1)	1.01 (0.98–1.05)	1.03 (1.0–1.06)	658 (69.5)	0.96 (0.90–1.02)	1.0 (0.94–1.06)	211 (48.6) *	0.84 (0.72–0.98) *	0.88 (0.77–1.0)
**No disability in hearing (Ref)**	1748 (26.5)	1	1	5683 (86.3)	1	1	4758 (72.2)	1	1	2215 (57.2)	1	1
**Any disability in hearing**	43 (35.1)	1.32 (0.94–1.87)	1.31 (0.95–1.81)	102 (83.2)	0.96 (0.86–1.08)	1.01 (0.90–1.13)	74 (60.7)	0.84 (0.67–1.05)	(0.91 (0.72–1.14)	22 (36.7) *	0.64 (0.41–1.0)	0.76 (0.50–1.15)
**No disability in vision (Ref)**	1634 (26.2)	1	1	5383 (86.2)	1	1	4511 (72.2)	1	1	2132 (57.2)	1	1
**Any disability in vision**	157 (33.6) *	1.28 (1.05–1.58) *	1.12 (0.92–1.36)	402 (86.2)	1.0 (0.95–1.06)	1.01 (0.96–1.07)	322 (68.9)	0.95 (0.87–1.04)	0.98 (0.90–1.07)	105 (50.9)	0.89 (0.73–1.08)	0.88 (0.75–1.05)
**No disability in communication (Ref)**	1781 (26.7)	1	1	5760 (86.4)	1	1	4815 (72.2)	1	1	2232 (57.0)	1	1
**Any disability in communication**	11 (24.2)	0.91 (0.38–2.19)	1.0 (0.41–2.40)	25 (56.3) ***	0.65 (0.45–0.95) *	0.71 (0.50–1.02)	18 (40.8) ***	0.56 (0.35–0.91) *	0.66 (0.42–1.05)	5 (29.0)	0.51 (0.20–1.34)	0.64 (0.25–1.62)
**No disability in walking (Ref)**	1671 (26.2)	1	1	5506 (86.2)	1	1	4595 (72.0)	1	1	2167 (57.0)	1	1
**Any disability in walking**	121 (37.2) **	1.42 (1.15–1.76) **	1.22 (1.0–1.51)	279 (86.0)	1.0 (0.94–1.05)	1.0 (0.94–1.05)	237 (73.0)	1.01 (0.93–1.11)	1.03 (0.5–1.12)	70 (53.4)	0.94 (0.74–1.19)	0.98 (0.78–1.24)
**No disability in cognition (Ref)**	1682 (26.3)	1	1	5513 (86.3)	1	1	4609 (72.2)	1	1	2171 (57.2)	1	1
**Any disability in cognition**	110 (33.9) *	1.29 (1.03–1.62) *	1.16 (0.93–1.44)	272 (84.1)	0.97 (0.92–1.03)	1.0 (0.95–1.05)	224 (69.1)	0.96 (0.86–1.07)	1.0 (0.92–1.10)	67 (47.0)	0.82 (0.63–1.05)	0.87 (0.70–1.09)
**No disability in self-care (Ref)**	1777 (26.7)	1	1	5728 (86.2)	1	1	4790 (72.1)	1	1	2226 (56.9)	1	1
**Any disability in self-care**	14 (21.6)	0.81 (0.39–1.69)	0.78 (0.37–1.64)	57 (87.5)	(0.87–1.18)	1.07 (0.92–1.24)	42 (65.3)	0.91 (0.70–1.18)	(0.79–1.27)	12 (50.0)	0.88 (0.47–1.63)	1.20 (0.67–2.11)

*** <0.001 ** <0.01 * <0.05

^1^Adjusted for age, education, wealth quintile, residence, and parity of the respondent.

Note: For skilled PNC, the study sample includes women aged 15–49 years giving birth in the 2 years preceding the survey. (n = 3,935)

A significantly higher proportion (86.4%) of women without any communication disability received ANC from a skilled provider as compared to the group with disabilities (56.3%) (p value <0.001). In bivariate analysis, women with some difficulty in communication had 35% lower chances of receiving skilled ANC (PR = 0.65; CI 0.45–0.95; p value <0.05). Likewise, SBA was also more prevalent (72.2%) in women who were able to communicate properly as compared to the women with disabilities (40.8%). However, the findings were insignificant in the adjusted model.

Women with disabilities had 16% lesser odds of receiving PNC from a skilled provider as compared to their counterparts (PR = 0.84; CI 0.72–0.98l p value <0.05). Although the results were rendered insignificant in the adjusted model but overall, the proportions of women receiving skilled PNC were consistently lower among the group with disabilities.

Overall, no significant difference was seen in the utilization of essential reproductive health services (modern contraceptive use, skilled ANC, SBA and skilled PNC) between women with and without disabilities when adjusted for important covariates.

The results of subgroup analysis are presented in Table 3. The p-values for the models that contain the full dataset with either the poor-rich or urban-rural interaction term with the disability measures are also reported in the table. With the exception of skilled PNC, none of the interaction term in the full model was found to be significant, which shows that the relationship between disability status and utilization of essential reproductive health services does not differ by wealth status and type of residence. The p-value for the poor-rich and urban-rural interaction in the skilled PNC model was significant, which indicated that the utilization of skilled PNC by women with any disability is dependent on their wealth status and type of residence.

**Table 3:** Adjusted prevalence ratio (APR) along with 95% CI for inequalities in the utilization of essential reproductive health services between women with and without disabilities, according to wealth quintile and place of residence. (n = 6,711).

	Wealth Status^1^					Place of residence				
	Poor		Rich		P-value for poor-rich difference	Urban		Rural		P-value for urban-rural difference
Variables	%	APR^2^ (95% CI)	%	APR^2^ (95% CI)		%	APR^3^ (95% CI)	%	APR^3^ (95% CI)	
**Modern contraceptive use**										
**No disability (Ref)**	22.4	1	31.1	1		30.1	1	23.7	1	
**Any disability in one or more domains**	30.5**	1.14 (0.95–1.36)	35.2	1.04 (0.80–1.35)	0.154	32.1	1.0 (0.74–1.23)	32.3**	1.20 (0.99–1.45)	0.766
**No severe disability (Ref)**	23.7	1	31.8	1		30.4	1	25.0	1	
**Any severe disability in one or more domains**	20.1	0.74 (0.45–1.22)	25.2	0.75 (0.41–1.40)	0.236	30.3	0.92 (0.53–1.60)	17.9	0.65 (0.37–1.16)	0.770
**Skilled ANC**										
**No disability (Ref)**	79.0	1	97.0	1		94.5	1	81.9	1	
**Any disability in one or more domains**	82.0	1.05 (1.0–1.10)	96.5	1.0 (0.97–1.03)	0.065	93.2	1.0 (0.96–1.03)	83.7	1.05 (1.0–1.10)	0.933
**No severe disability (Ref)**	79.4	1	96.9	1		94.2	1	82.1	1	
**Any severe disability in one or more domains**	79.8	1.03 (0.91–1.17)	99.7**	1.04 (1.02–1.06) ***	0.612	95.8	1.03 (0.98–1.09)	81.6	1.03 (0.92–1.16)	0.152
**Skilled birth attendant**										
**No disability (Ref)**	61.4	1	89.5	1		86.0	1	65.7	1	
**Any disability in one or more domains**	59.1	0.98 (0.89–1.08)	88.4	1.0 (0.96–1.07)	0.667	80.8	0.96 (0.89–1.03)	63.2	1.01 (0.94–1.10)	0.298
**No severe disability (Ref)**	61.3	1	89.3	1		85.4	1	65.5	1	
**Any severe disability in one or more domains**	54.6	0.92 (0.74–1.15)	92.3	1.08 (0.98–1.18)	0.495	80.1	0.97 (0.84–1.13)	60.4	0.97 (0.80–1.18)	0.744
**Skilled PNC**										
**No disability (Ref)**	44.8	1	78.8	1		74.1	1	50.1	1	
**Any disability in one or more domains**	34.5**	0.77 (0.62–0.97) *	76.6	1.0 (0.88–1.13)	0.001**	64.4	0.91 (0.76–1.07)	39.7	0.85 (0.69–1.04)	0.044*
**No severe disability (Ref)**	43.9	1	78.8	1		73.2	1	49.3	1	
**Any severe disability in one or more domains**	31.1	0.75 (0.42–1.33)	66.2	1.0 (0.66–1.44)	0.133	58.5	0.93 (0.60–1.43)	32.2	0.76 (0.42–1.36)	0.259

*** <0.001 ** <0.01 * <0.05

^1^Poor includes “poorest”, “poorer” and “middle” category, while rich includes “richer” and richest”

^2^Adjusted for age, education, residence, and parity.

^**3**^Adjusted for age, education, wealth quintile, and parity.

Note: For skilled PNC, the study sample includes women aged 15–49 years giving birth in the 2 years preceding the survey. (n = 3,935)

The APR given in the table are derived from the regression model after adjusting for the covariates. Most of the APR were insignificant, the only exception was skilled ANC, where women with severe disability in at least one domain were 1.04 times more likely to get ANC from a skilled provider if they belonged to the rich socioeconomic class (APR = 1.04; CI 1.02–1.06; p value <0.001). A weakly negative association was also seen among the poor-rich socioeconomic group, where women with disabilities belonging to the poor class were 23% less likely to receive PNC from a skilled health professional (APR = 0.77; CI 0.62–0.97; p value <0.05).

## Discussion

Our current analyses provide key insights into disability patterns and their association with maternal and reproductive health services utilization among women of childbearing age in Pakistan. To the best of our knowledge, this study is one of the first to provide information about disparities in the utilization of key maternal and reproductive health services to women with disabilities in Pakistan. Overall, utilization of essential maternal and reproductive health services was not different between women with and without disability.

### Socio-demographic differentials

According to the World Report on Disability, the global prevalence of any form of disability among women was reported to be 19.2% [1]. In our study, women with disabilities comprised nearly 14% of the total study sample. These estimates were slightly higher than that reported by a multi-country study from Africa, where the prevalence of disability ranged between 6.0–8.0% among women aged 15–39 years [32]. Our results were somewhat similar to the findings from a nationally representative data set of Bangladesh who reported a disability prevalence of nearly 11.0% among female household members [33].

The three most common types of disability reported worldwide are physical disability, visual impairment, and hearing disability [1]. In our study, the most prevalent domains were vision, walking, and cognition, whereas, disability in self-care and communication were least commonly reported. Our findings are consistent with studies conducted in Ghana and Nepal, where physical and visual impairments were among the top three most commonly cited disabilities by women of reproductive age group [34, 35].

Data from 59 countries have found that the average disability prevalence rates are higher among individuals belonging to the older age group and lower wealth quintiles [1]. We also found some evidence of socio-demographic disparity in our sampled population with significantly higher proportions of older and less educated women among the group with disabilities as compared to their non-disabled counterparts. In India, none of the women with disabilities had been educated to graduation or beyond as compared to the non-disabled group [36]. Likewise, majority of women (58.2%) with disabilities in Nepal were of higher age (30–49 years) and had a lower literacy rate (59.5%) than women without disabilities (70.0%) [35]. Similar findings were also reported from England’s National Health Survey [37].

In contrast to other studies, we found no significant difference in the prevalence of disability with respect to wealth status and type of residence [1, 33]. Literature from different regions of the world exploring disparities in the distribution of disabilities has reported mixed findings in this regard. A study from Bangladesh found that people belonging to middle and rich socio-economic class had a 14 percent lower likelihood of reporting disabilities than poor families [33]. Literature from the United States (US) and Nepal has documented a higher prevalence of disability in rural settings, whereas, opposite was true in case of Tanzania [32, 35, 38]. Since our analysis was primarily focused on women of childbearing age, thus the results may not be generalizable to other segments of the population and comparison should be made with caution. However, our finding of no socio-economic disparity in the utilization of essential reproductive health services with respect to disability status of women is consistent with that reported by Waqas et al. who also found that the utilization of antenatal services by women with disabilities is not different than the non-disabled women regardless of their wealth status and type of residence [27].

While it is widely assumed that disability and wealth status are closely associated, these links are rather complex, multidimensional, and nuanced than is currently presumed. A critical review of the literature exploring the relationship between poverty and disability in LMICs suggested a lack of strong evidence on the links between the two indicators [39]. Both disability and wealth status of a household are difficult to define and measure due to their multifaceted nature and lack of universally acceptable definitions [1, 40]. Even in national censuses and large demographic and health surveys, disability is assessed only through a limited number of questions that diminishes their utility and cross comparability [1]. As emphasized by Braithwaite and Mont, these methodological challenges in measuring disability consequently effect the associations found between disability and different socioeconomic characteristics [41]. In order to establish a causal association between disability and various socioeconomic factors, there is a need to conduct longitudinal studies to have more robust data.

In addition, we found a significantly higher proportion of women with disabilities having more than three living children compared to non-disabled women. Our finding is comparable with that reported from India [36]. One of the reasons cited in the literature for higher parity among women with disabilities is decreased use of contraceptives amongst them [42]. In our study, although the current use of modern contraceptives was more prevalent among the group with disabilities, a higher number of living children is possibly a consequence of decreased utilization in the past. Nevertheless, data regarding previous contraceptive practices are required to support this postulation. Another reason for this difference could be that a higher proportion of women with disabilities in our sample belonged to the older age group, hence this might explain more number of children in this subset as compared to their non-disabled counterparts who are young and yet to bear more children. Nonetheless, longitudinal data on disability and childbearing is required to establish a better understanding of this relationship.

### Disability and utilization of essential maternal and reproductive health services

In our descriptive analysis, we found the frequency of skilled ANC and modern contraceptive use to be either comparable or higher among women with disabilities, respectively. According to the literature, women with disabilities often experience pressure from their families and community not to have children [43]. They are in general discouraged from pregnancy out of misplaced fear that their children will in turn have disabilities or their pregnancy might lead to unwarranted complications [44]. A study from the US reported that women with disabilities were significantly less likely to intend to have more children than other women [45]. These factor could possibly contribute to an increased contraceptive use (or in extreme cases, forced sterilization) among women with disabilities to delay or limit childbearing. It is also important to mention about the Lady Health Worker (LHW) Program of Pakistan, which is one of the largest public health program of the country comprising more than 100,000 LHWs who provide maternal and child healthcare services through regular door to door visits, especially in hard to reach areas [46]. These services include contraception, antenatal care, iron and folic acid supplementation during pregnancy, growth monitoring of children, and counselling for vaccination of mothers and children. The main purpose of providing these home-based services is to minimize the barriers related to transportation and cost in accessing these services, which are also commonly faced by the people with disabilities [8]. The fact that we found no difference in the utilization of skilled ANC among both groups, and a higher prevalence of modern contraceptive use in women with disabilities could also be partly attributed to the active involvement of LHWs in the provision of these services during their routine household visits.

Although literature from both developed and developing parts of the world has provided some indication on the link between disability and inequitable distribution of healthcare services, but our analysis could not generate sufficient evidence to support this notion [1, 47, 48]. We found no significant difference in the utilization of essential maternal and reproductive health services between women with and without disabilities after adjusting for important covariates. A systematic review assessing access to general healthcare services for people with disabilities in LMICs also reported no difference in indicators of maternal health coverage for women with disabilities [8]. However, the findings of the systematic review should be interpreted with caution since the outcomes evaluated were not uniform across various studies. Similar results were reported from India and the United Kingdom (UK), where reproductive health parameters such as pregnancy outcome, timing of first ANC contact, utilization of ANC and PNC services, and post-partum mid-wife contacts and contraceptive counseling were either comparable or higher among the group with disabilities as compared to the non-disabled women [36, 37]. Another matched cohort study from the US documented a significantly higher proportion of women with intellectual disabilities utilizing outpatient and home health services both in the early and late post-partum period [49]. Although these findings are encouraging and reflect better responsiveness of the healthcare system towards socially disadvantaged people, efforts are still needed to improve the quality of services to account for the different needs of individuals with disabilities [50].

Despite the fact that the utilization of healthcare services by women with disabilities has shown some improvement over time, still they experience disparities in the quality of care [51, 52]. A qualitative survey conducted in Ghana reported an increased desire for childbearing and skilled care among women with disabilities. However, HCW’s insensitive behavior and lack of knowledge regarding maternity care needs of women with disabilities served as major challenges in accessing these services [34]. Similarly, a review article indicated increased utilization of healthcare services by women with disability [53]. The fact that we have also found women with disabilities utilizing essential maternal and reproductive health services at a comparable frequency in a way indicates that maternity care in Pakistan has begun to match the needs of women with disabilities to some extent. Nevertheless, it is yet to be explored how well the health system is able to respond to the different psychosocial needs of women with disabilities throughout the continuum of care. Additionally, a higher parity seen among women with disabilities in our study also emphasizes the need to reexamine the widely held misperception that women with disabilities are sexually inactive and are less capable of childbearing. Based on our findings, it is critical to realize that women with disabilities also possess the same biological and reproductive needs as any other women in the community. Therefore, this calls for action to allocate more resources for developing disability-friendly healthcare services, and for providing disability-related cultural competence training to healthcare professionals to optimize the quality of services provided to this vulnerable segment of the population [34].

### Strengths and limitations

The current analysis is based on the most recent national survey of Pakistan. The results of this study add important information to the growing body of evidence on maternal and reproductive health care delivery patterns among women with disabilities in LMICs. This was a comprehensive analysis defining pattern of health disparities between women with and without disability in four major maternal and reproductive health areas of public health significance. Hence, this would allow greater comparability of outcomes across countries. In addition, the survey utilized a validated tool to assess functional disability among the study sample. Lastly, a sub-group analysis was also performed to assess the role of wealth status and type of residence as effect modifiers in the relationship between disability and utilization of essential maternal and reproductive health services.

This study has a few limitations. Since this was a cross-sectional analysis hence the associations established cannot be considered as causal. In DHS surveys, data on disability in collected from the household head who provides information for all the household members, hence the chances of information bias are high. Additionally, although the information collected from the woman is regarding her most recent birth, recollection of reproductive history could still lead to recall bias. Also, for most of the reproductive health indicators, DHS sample is restricted to only those women who had a live birth in the five years before the survey, hence, some of the information dating back to five years might not be captured in DHS surveys. Secondly, since DHS surveys employ only quantitative questionnaire, hence the influence of social factors such as stigmatization and discrimination against group with disabilities could not be explored in this study which are better understood through a qualitative approach. Last but not the least, this study provides important evidence regarding crude measures of utilization of essential maternal and reproductive health services, however, we did not take into account the quality of care aspect for women with disabilities in Pakistan which should be explored in future.

## Conclusion

The study demonstrates that women with disability are significantly more likely to have higher number of children and lower education status. In contrast to previous literature, our analyses did not find any statistically significant differences in the utilization of essential maternal and reproductive health services between women with and without disabilities. There is a need to undertake further research that could help us understand how well the healthcare system in Pakistan is responsive to the differential needs of women with disabilities. We recommend use of mixed methods design in future to explore challenges women with disabilities face in terms of affordability, accessibility and quality of maternal health care services being provided to them. Moreover, in order to make SRH services more universal and inclusive of people with varying social characteristics, it is pivotal to study the numerous and coexisting intersectional vulnerabilities people with different types of disabilities are subjected to.
